# Estimating Carbon Flux Phenology with Satellite-Derived Land Surface Phenology and Climate Drivers for Different Biomes: A Synthesis of AmeriFlux Observations

**DOI:** 10.1371/journal.pone.0084990

**Published:** 2013-12-27

**Authors:** Wenquan Zhu, Guangsheng Chen, Nan Jiang, Jianhong Liu, Minjie Mou

**Affiliations:** 1 State Key Laboratory of Earth Surface Processes and Resource Ecology, Beijing Normal University, Beijing, China; 2 College of Resources Science and Technology, Beijing Normal University, Beijing, China; 3 Environmental Sciences Division, Oak Ridge National Laboratory, Oak Ridge, Tennessee, United States of America; Cirad, France

## Abstract

Carbon Flux Phenology (CFP) can affect the interannual variation in Net Ecosystem Exchange (NEE) of carbon between terrestrial ecosystems and the atmosphere. In this study, we proposed a methodology to estimate CFP metrics with satellite-derived Land Surface Phenology (LSP) metrics and climate drivers for 4 biomes (i.e., deciduous broadleaf forest, evergreen needleleaf forest, grasslands and croplands), using 159 site-years of NEE and climate data from 32 AmeriFlux sites and MODIS vegetation index time-series data. LSP metrics combined with optimal climate drivers can explain the variability in Start of Carbon Uptake (SCU) by more than 70% and End of Carbon Uptake (ECU) by more than 60%. The Root Mean Square Error (RMSE) of the estimations was within 8.5 days for both SCU and ECU. The estimation performance for this methodology was primarily dependent on the optimal combination of the LSP retrieval methods, the explanatory climate drivers, the biome types, and the specific CFP metric. This methodology has a potential for allowing extrapolation of CFP metrics for biomes with a distinct and detectable seasonal cycle over large areas, based on synoptic multi-temporal optical satellite data and climate data.

## Introduction

Vegetation phenology plays an important role in adjusting the annual Net Ecosystem Exchange (NEE) (see Acronym S1 in supporting information for a list of acronyms and definitions used in this paper) of carbon between terrestrial ecosystems and the atmosphere [1]–[5]. The interannual variation in ecosystem productivity caused by vegetation phenology shifts was widely investigated by field studies [6]–[9] and ecosystem models [10]–[14]. An earlier start or/and a later end of vegetation growing season can extend the period of photosynthesis, and thus increased primary productivity is expected. Indeed, some previous studies have shown a positive effect of Growing Season Length (GSL) on net productivity (e.g., 5.9 g C•m^−2^•d^−1^ in a deciduous forest [15] and around 4 g C•m^−2^•d^−1^ in a subtropical forest stand [16]). Moreover, the length of Carbon Uptake Period (CUP) has much predictive power about the spatial variation of annual NEE. For example, the length of CUP can explain 80% of the spatial variance in annual NEE for deciduous forests across a latitudinal and continental gradient [17].

There are currently numerous data sources available for estimating the timing of recurrent vegetation phenology transitions, such as the ground-, satellite- and eddy covariance flux-based data sources [18]. Land Surface Phenology (LSP) is defined as the study of the timing of recurring seasonal pattern of variation in vegetated land surfaces observed from synoptic sensors [19], [20]. Satellite-based LSP is characterized by the Start (SOS) and End (EOS) of growing Season, which are closely related to vegetation growth or photosynthesis. Carbon Flux Phenology (CFP) is defined as the detrended zero-crossing timing of NEE from a source to a sink in spring and *vice versa* in autumn [3], [4], [18], [19]. CFP is characterized by the Start (SCU) and End (ECU) of Carbon Uptake, which are closely related to the difference between growth and respiration. LSP allows the determination of GSL or the duration of canopy coverage from the difference between EOS and SOS, while CFP allows the determination of CUP from the difference between ECU and SCU. The CUP is controlled by GSL, but is not identical because growth will typically commence and terminate some time before and after the NEE changes sign in spring and autumn, respectively [19], [21]. White & Nemani [13] found that there was a strong relationship between NEE and CUP, but a very weak relationship between NEE and GSL for deciduous forests. Thus, CUP is a potentially useful indicator of annual carbon sequestration [3]. However, the application of CUP is hindered by the limited number of flux towers and the distribution and footprint of these flux towers [3], [19], [21]. Although more than 500 tower sites from approximately 30 regional networks across 5 continents are currently operating on a long-term basis, these globally distributed eddy flux sites sample only a small subset of the Earth's biomes, disturbance regimes, and land management systems. Thus, estimation of CUP over large areas remains challenging [19], [21], [22].

Some limited attempts have been made to estimate CFP dates beyond the footprints of flux towers [18], [19], [21], [22]. Using over 30 site-years of data from 12 eddy flux sites, Baldocchi *et al*. [22] found that 64% of variance in SCU can be explained by the date when soil temperature matched the mean annual air temperature. Remote sensing provides spatially comprehensive measures of ecosystem activity and therefore is a potentially powerful tool to allow extrapolation of CUP over large areas. To test the capabilities of remote observations in estimating CUP, Churkina *et al*. [21] related the GSL from remotely sensed data to the CUP from eddy flux tower measurements and found a strong relationship between them. However, a comparison of multiple phenology data sources indicated that no single source of phenological data was able to accurately describe annual patterns of flux phenology [18]. Therefore, Gonsamo et al. [19] combined LSP dates with the mean monthly surface temperature derived from remote sensing observations to predict CUP. Their results indicated that remote sensing-derived multiple surface variables can explain CUP variability by more than 70% in spring and autumn. However, this CUP determination approach is just based on four selected temperate and boreal deciduous forest CO_2_ flux tower sites. A more comprehensive analysis, based on multi-year data from eddy flux sites across large areas for various biome types, is still expected. Moreover, improved estimation of LSP dates combined with optimal climate drivers may further enhance the CUP estimation performance.

Using data from a large number of AmeriFlux sites, this study aims to estimate CFP metrics with satellite-derived LSP metrics and climate drivers for different biomes, including deciduous broadleaf forest, evergreen needleleaf forest, grasslands and croplands. We first evaluated different LSP retrieval methods and Vegetation Index (VI) products based on the observed CFP dates and selected the best performing method and VI product to retrieve LSP dates as the explanatory variables in estimating both SCU and ECU. Then, we carried out a sensitive analysis to search the optimal explanatory climate drivers for the estimation of SCU and ECU. Finally, the estimated LSP dates and the selected optimal explanatory climate drivers were combined to estimate CFP dates, and a comprehensive discussion was given to highlight the limitations and potentials of the proposed methodology.

## Data and methods

### Data and pre-processing

#### Site carbon flux and meteorological data

**Figure 1:**
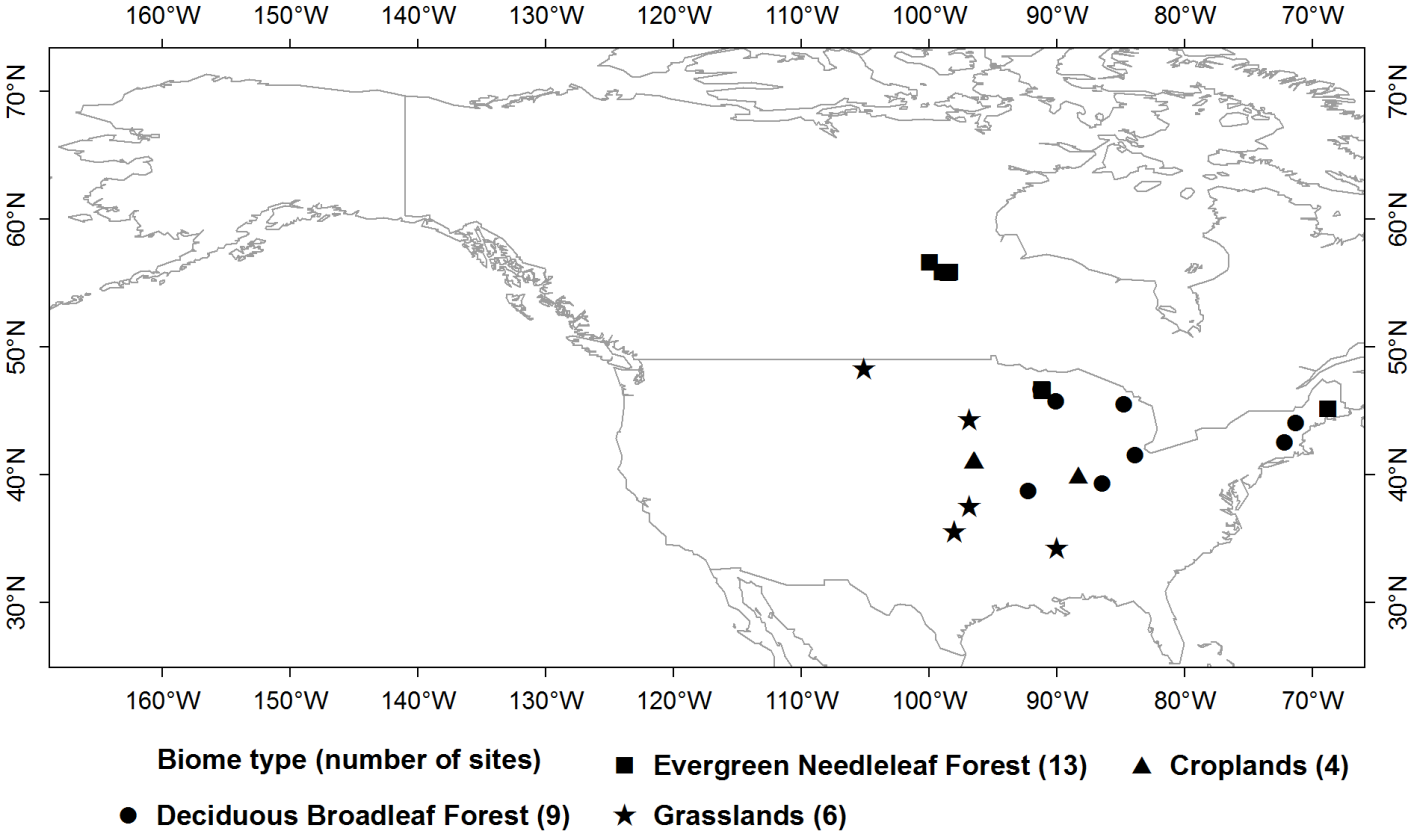
Distribution of eddy flux towers and their corresponding biome types.

The daily NEE (g C•m^−2^•d^−1^), air temperature (°C) and precipitation (mm) data used in this study were derived from the post-processed Level 4 product (available at: http://daac.ornl.gov/FLUXNET/fluxnet.shtml) of the AmeriFlux sites (Figure 1, Dataset S1). The covered period for the product was generally from 1995 to 2007 but depending on the specific site. For example, the acquired NEE and meteorological data were from 1998 to 2007 for the Niwot Ridge site, while they were from 1995 to 1999 for the Walker Branch site in the United States. The same years for having both NEE and Moderate Resolution Imaging Spectroradiometer (MODIS) VI data were used for analysis. Therefore, our analysis only focused on the period of 2000–2007 since the overlay time period for both data sets only covered from February 2000 (Start date for MODIS VI data) to December 2007 (End date for available NEE data). For each biome type, we first excluded the sites with more than 60 days deviations from the average SCU and ECU. We regarded each year for each flux tower site as one site-year and excluded the site-years whose daily NEE values were missing for the carbon source-sink or sink-source transition period. Moreover, only the biomes with at least 10 site-years were included for analysis in order to get robust estimations for CFP dates. Therefore, we got 32 eddy flux sites, which covered 4 biome types according to the International Geosphere-Biosphere Program (IGBP) classification system (Figure 1). There were totally 73 site-years involved in the spring carbon source-sink transition period and 86 site-years involved in the autumn carbon sink-source transition period.

Remotely sensed data. The Terra's MODIS 250 m 16-day composited VI products (MOD13Q1, V005) for the 32 flux tower sites were used in this analysis (available at: http://daac.ornl.gov/MODIS/). The first VI product was the standard Normalized Difference Vegetation Index (NDVI), and the second was the Enhance Vegetation Index (EVI). The VI time series for the pixel located at the center of the flux tower was used to retrieve land surface phenological metrics. The covered period for the VI data was the same as the NEE data for a given flux site. Much noise existed in the VI time series because of cloud contamination, atmospheric variability and sun-sensor-surface viewing geometries [23], [24]. A filtering process was needed before using VI to retrieve phenological metrics [25]. We used the Savitzky-Golay filter method to remove the noise in the VI time series [26].

### Methods

#### Retrieving CFP dates from NEE data

**Figure 2:**
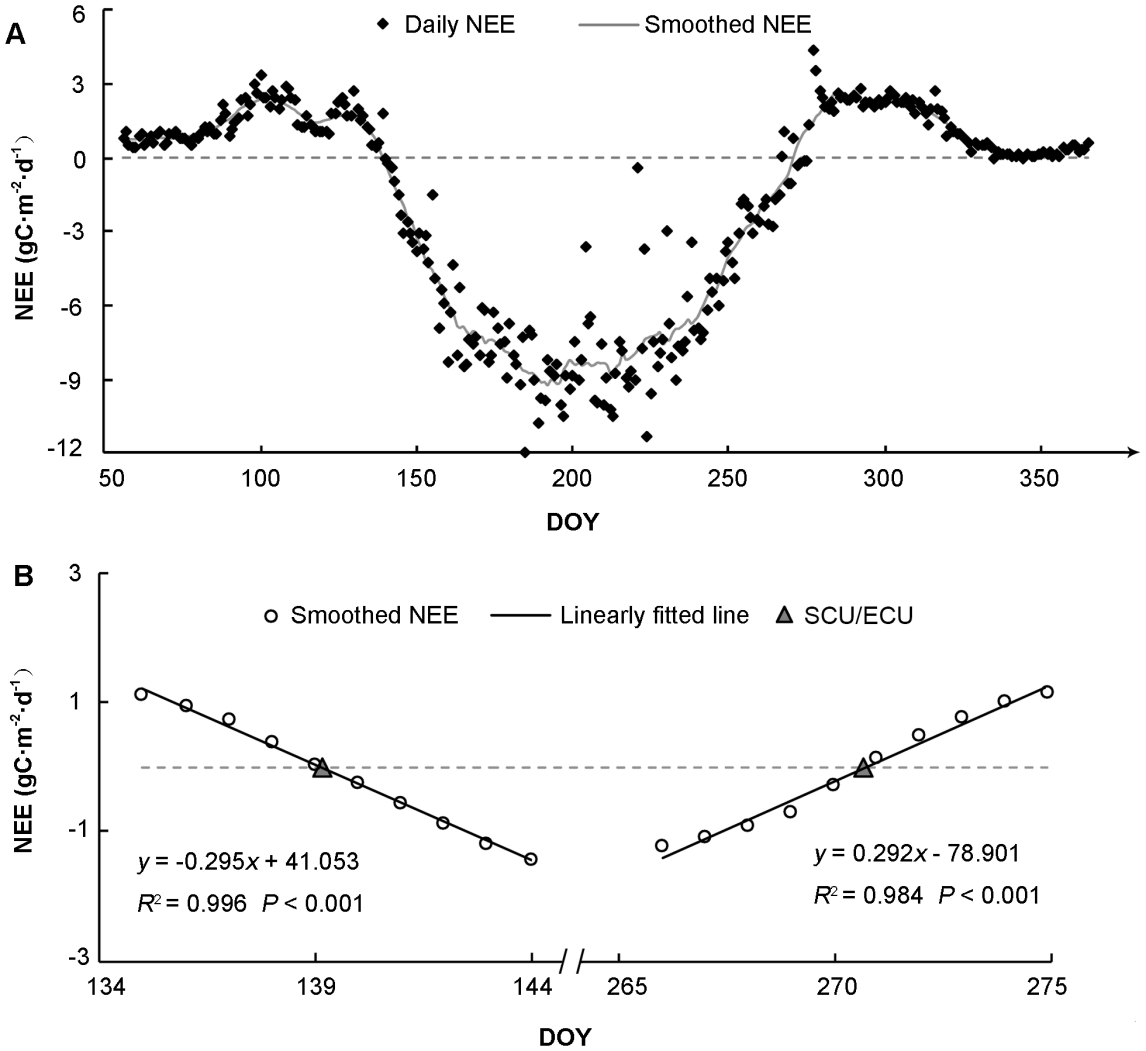
A schematic demonstration of the retrieval method for carbon flux phenology dates. A) The original and smoothed 15-day mean Net Ecosystem Exchange (NEE) of carbon, and B) the two selected transition periods for spring source-sink and autumn sink-source for identifying linear regressions between NEE and the Julian Day of Year (DOY). Start/End of Carbon Uptake (SCU/ECU) is estimated at the zero intersection.

The SCU and ECU were retrieved based on the method proposed by Baldocchi *et al*. [22]. The original method is based on visual interpretation of the daily NEE time series. We developed this method to retrieve SCU and ECU automatically through fitting a regression equation between the daily NEE and the Julian Day of Year (DOY), using subsets of NEE data from spring source-sink and autumn sink-source transition periods, respectively (Figure 2). Specifically, SCU and ECU were automatically retrieved by the following three steps: (1) the original daily NEE was smoothed with a moving average of a 15-day width; (2) based on the smoothed daily NEE, a 10-day width window with the first 5 elements greater than zero and the last 5 elements less than zero was selected in the spring/summer period to predict SCU, and another 10-day width window with the first 5 elements less than zero and the last 5 elements greater than zero was selected in the autumn/winter period to predict ECU; and (3) the smoothed daily NEE in the two selected windows was linearly regressed to predict SCU and ECU at the zero intersection.

#### Retrieving LSP dates from remotely sensed data

A number of methods have been developed to retrieve land surface phenology metrics using satellite VI time series [27]–[29]. These methods can be classified into 3 types: the threshold method (i.e., a global absolute threshold value or a local relative threshold value defined as a fraction of the annual amplitude) [30]–[33], the autoregressive moving average method [27], [34] and the function fitting method [28], [35]–[39]. Almost all the methods mentioned above have been proven to be consistent with their given references (e.g., ground observed phenology events, model simulated vegetation phenology or eddy covariance flux tower-derived phenological metrics), but it was very difficult to give the ordinal rank of SOS methods because they varied geographically [27]. Therefore, this study first investigated these 3 types of satellite methods (including 6 specific retrieval methods) based on the first MODIS VI product (i.e., NDVI), and selected the one with the best performance to retrieve LSP dates. Then, a comparison between the two MODIS VI products (i.e., NDVI and EVI) was carried out based on the best-performing LSP retrieval method, and the more suitable VI product was selected to retrieve LSP dates as the explanatory variables in estimating CFP dates. The detailed descriptions about these 6 retrieval methods and the evaluation process were given in Text S1.

#### Identifying the explanatory climate drivers

**Figure 3:**
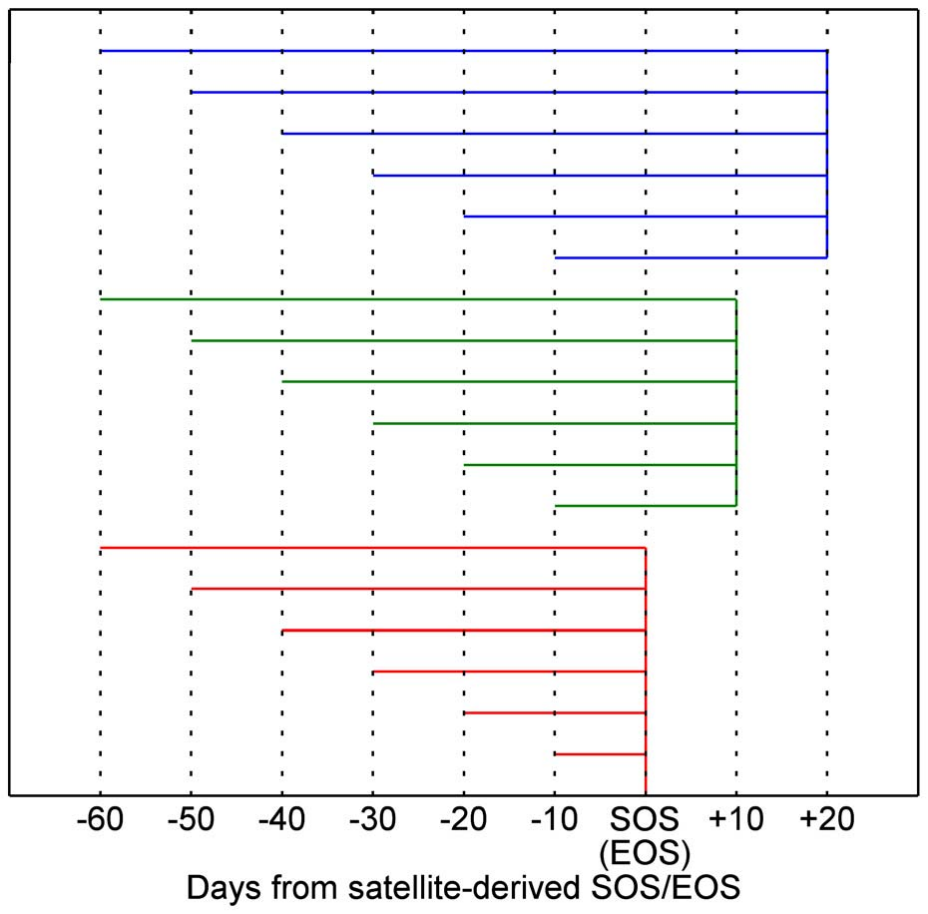
A schematic representation of the different impact periods for climate drivers. The different impact periods of climate drivers on carbon flux phenology dates were determined in terms of the distance (in days) from satellite-derived Start/End of Season (SOS/EOS), 10-day after SOS/EOS and 20-day after SOS/EOS with a step of 10 days. There were totally 18 candidate impact periods for each climate driver. Negative values indicate the days before SOS/EOS and positive values indicates the days after SOS/EOS.

The LSP dates derived from the more suitable VI product with the best satellite retrieval method, the cumulative daily air temperature (above 0°C) and total precipitation were used to identify the explanatory climate drivers. Previous studies indicated that SCU usually occurs 0–20 days later than SOS while ECU usually occurs 0–60 days earlier than EOS [4], [19], [21]. Therefore, we restricted the impact period of climate drivers on SCU/ECU in the range from 60 days before SOS/EOS to 20 days after SOS/EOS. To identify the optimal impact period for each climate driver, different impact periods were tested according to the distance (in days) from SOS/EOS, 10-day after SOS/EOS and 20-day after SOS/EOS with a step of 10 days (Figure 3). Therefore, we got 18 candidate impact periods for each climate driver (i.e., cumulative daily air temperature (above 0°C) or total precipitation). The coefficient of determination (*R* ^2^) between observed SCU/ECU and each climate driver with different candidate impact periods was used to select the best explanatory climate drivers. Only the climate driver in a given impact period with the highest *R* ^2^ in its group (i.e., 18 candidate cumulative temperature or total precipitation data for each biome type and each phenological metric) and with a statistical significance at the 0.05 level will be selected to estimate SCU/ECU.

#### Estimating CFP dates

Using the least-squares linear regression model, the CFP dates (i.e., SCU and ECU) can be estimated with the LSP dates (i.e., SOS and EOS) and the selected explanatory climate drivers. The estimating performance of the linear regression models was evaluated with coefficient of determination (*R* ^2^), Root Mean Square Error (RMSE) and the leave-one-out cross-validation approach [19], [40]. Significance test for these linear regression models was conducted by *F*-test with the standard 0.05 cutoff indicating statistical significance (i.e., *P*<0.05).

## Results

### The relationship between CFP and LSP dates

Our evaluation about the different LSP retrieval methods and MODIS VI products indicated that the NDVI-derived LSP dates with the local mean midpoint threshold method were more consistent with the observed CFP dates (see details in Text S2). Table 1 showed the relationship between CFP and LSP dates. SOS explained the SCU variance by 43.1%–78.4% for different biomes. The RMSE between SCU and SOS ranged from 2.7 to 7.6 days, which was far smaller than the temporal resolution of the satellite data (∼16 days). This indicated that the SCU can be estimated with SOS to the accuracy that was comparable to the 16-day composited temporal resolution of satellite sensor. Comparing with the SCU, lower performance was found for estimating ECU based on EOS, with relatively lower explanatory variances and higher RMSE for different biomes. However, this RMSE was still comparable with the 16-day composited temporal sampling resolution of satellite data.

**Table 1:** The coefficient of determination (*R* ^2^), Root Mean Square Error (RMSE) and Bias between Net Ecosystem Exchange (NEE)-derived carbon flux phenology dates and Normalized Difference Vegetation Index (NDVI)-derived land surface phenology dates based on the best performing retrieval method (i.e., the local mean midpoint threshold method) for different biomes.

Biome type	SOS vs. SCU†				EOS vs. ECU†			
	Samples	*R* ^2^ (%)	RMSE	Bias	Samples	*R* ^2^ (%)	RMSE	Bias
Deciduous broadleaf forest	24	74.3*	7.5	−10.5	20	51.4*	6.3	4.6
Evergreen needleleaf forest	16	78.4*	7.6	15.8	30	43.5*	13.1	35.6
Grasslands	16	43.1*	6.5	3.3	14	67.1*	10.3	9.4
Croplands	17	68.8*	2.7	−0.2	22	65.0*	5.6	14.7
All biomes	73	49.6*	17.1	0.7	86	43.5*	14.6	18.8

SOS = Start of Season derived from satellite data, SCU = Start of Carbon Uptake derived from carbon flux data, EOS = End of Season derived from satellite data, ECU = End of Carbon Uptake derived from carbon flux data.

Statistically significant at the 0.05 level.

Different biomes showed distinctive CFP dates as estimated based on the LSP dates (Table 1). For example, evergreen needleleaf forest had the highest explanatory variance (78.4%) in estimating SCU based on SOS while grasslands had the lowest (43.1%). On the contrary, evergreen needleleaf forest showed the poorest performance in estimating ECU based on EOS while grasslands showed the best performance (67.1%). In general, the performance in estimating CFP dates for a single biome was better than multiple biomes.

### The relationship between CFP dates and climate drivers

**Figure 4:**
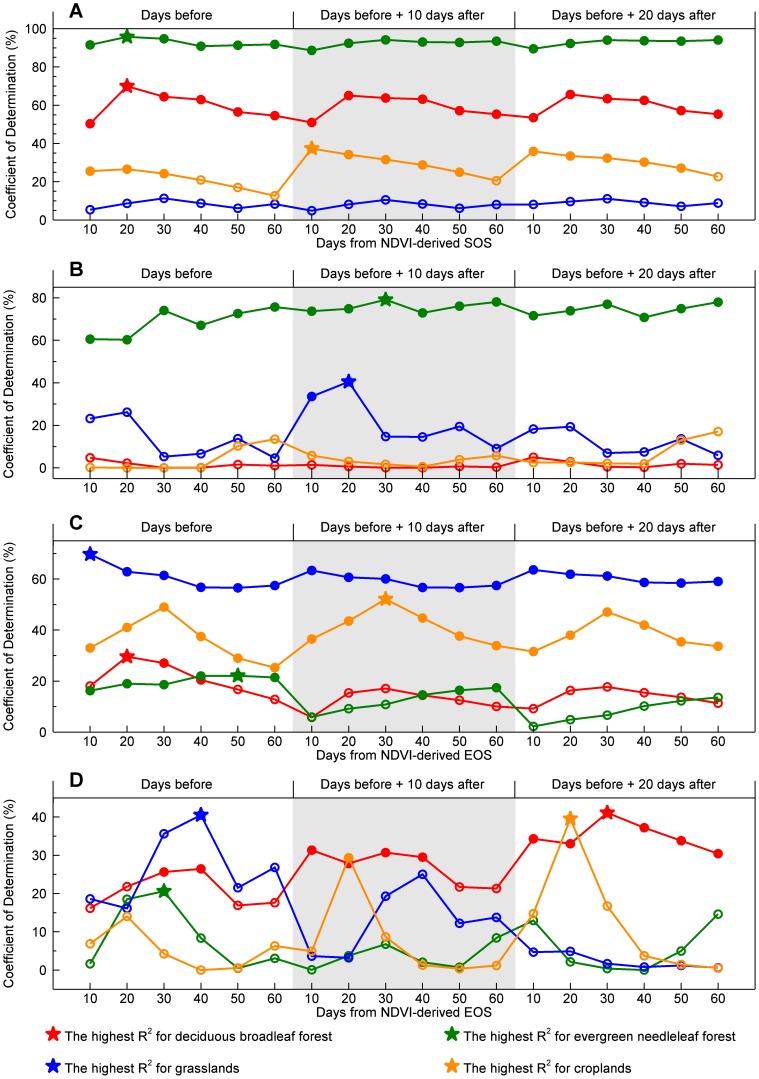
The relationships between carbon flux phenology dates and climate drivers in different impact periods. A) The coefficient of determination (*R* ^2^) between Net Ecosystem Exchange (NEE)-derived Start of Carbon Uptake (SCU) and the cumulative daily air temperature (above 0°C) for different periods around Normalized Difference Vegetation Index (NDVI)-derived Start of Season (SOS). B) The *R* ^2^ between NEE-derived SCU and the total precipitation for different periods around NDVI-derived SOS. C) The *R* ^2^ between NEE-derived End of Carbon Uptake (ECU) and the cumulative daily air temperature (above 0°C) for different periods around NDVI-derived End of Season (EOS). D) The *R* ^2^ between NEE-derived ECU and the total precipitation for different periods around NDVI-derived EOS. Red colored line: Deciduous broadleaf forest; green: evergreen needleleaf forest; blue: grassland; orange: cropland. Stars indicate the locations with the highest *R* ^2^ for each biome and with a statistical significance at the 0.05 level. Solid circles indicate statistically significant *R* ^2^ at the 0.05 level, and hollow circles indicate statistically non-significant *R* ^2^.

Figure 4 showed the coefficient of determination (*R* ^2^) between CFP dates and climate drivers during different impact periods. For SCU explanatory variances by the cumulative daily air temperature (above 0°C) (Figure 4A), evergreen needleleaf forest demonstrated better performance than other 3 biomes with consistently the highest *R* ^2^ values (ranging from 88.6% to 95.7% among the 18 tested impact periods). Considering its high explained variances, we selected the cumulative daily air temperature above 0°C during 20 days before NDVI-derived SOS as one of the explanatory variables in estimating SCU. Similarly, the total precipitation during the period of 30-day before SOS and 10-day after SOS was also selected for evergreen needleleaf forest in estimating SCU (Figure 4B). All the total precipitation variables during different impact periods were not significant at the 0.05 level for both deciduous broadleaf forest and croplands (Figure 4B). Therefore, no precipitation variables were selected for these two biomes to estimate SCU. In summary, the optimal impact periods for different climate drivers (i.e., cumulative air temperature and total precipitation) and different CFP metrics (i.e., SCU and ECU) were marked with stars in Figure 4. Only the climate drivers being significant during their impact periods were selected as the explanatory variables to estimate CFP dates.

The sensitivity of CFP metrics to climate drivers varied among different biomes (Figure 4). For SCU, evergreen needleleaf forest showed higher sensitivities to both cumulative temperature (above 0°C) and total precipitation, while deciduous broadleaf forest and cropland were only sensitive to cumulative temperature (Figure 4 A, B). Grassland had a higher sensitivity to total precipitation during the period of 20-day before SOS and 10-day after SOS but a lower sensitivity to cumulative temperature for the 6 selected grassland sites. For ECU, herbaceous biomes (i.e., grasslands and croplands) showed a higher sensitivity to cumulative temperature than woody biomes (e.g., deciduous broadleaf and evergreen needleleaf forest) (Figure 4 C). Deciduous broadleaf forest showed higher explained variances by total precipitation variables in different impact periods for ECU, while evergreen needleleaf forest demonstrated lower explained variances (Figure 4 D).

### Estimation of CFP dates with LSP dates and climate drivers

**Figure 5:**
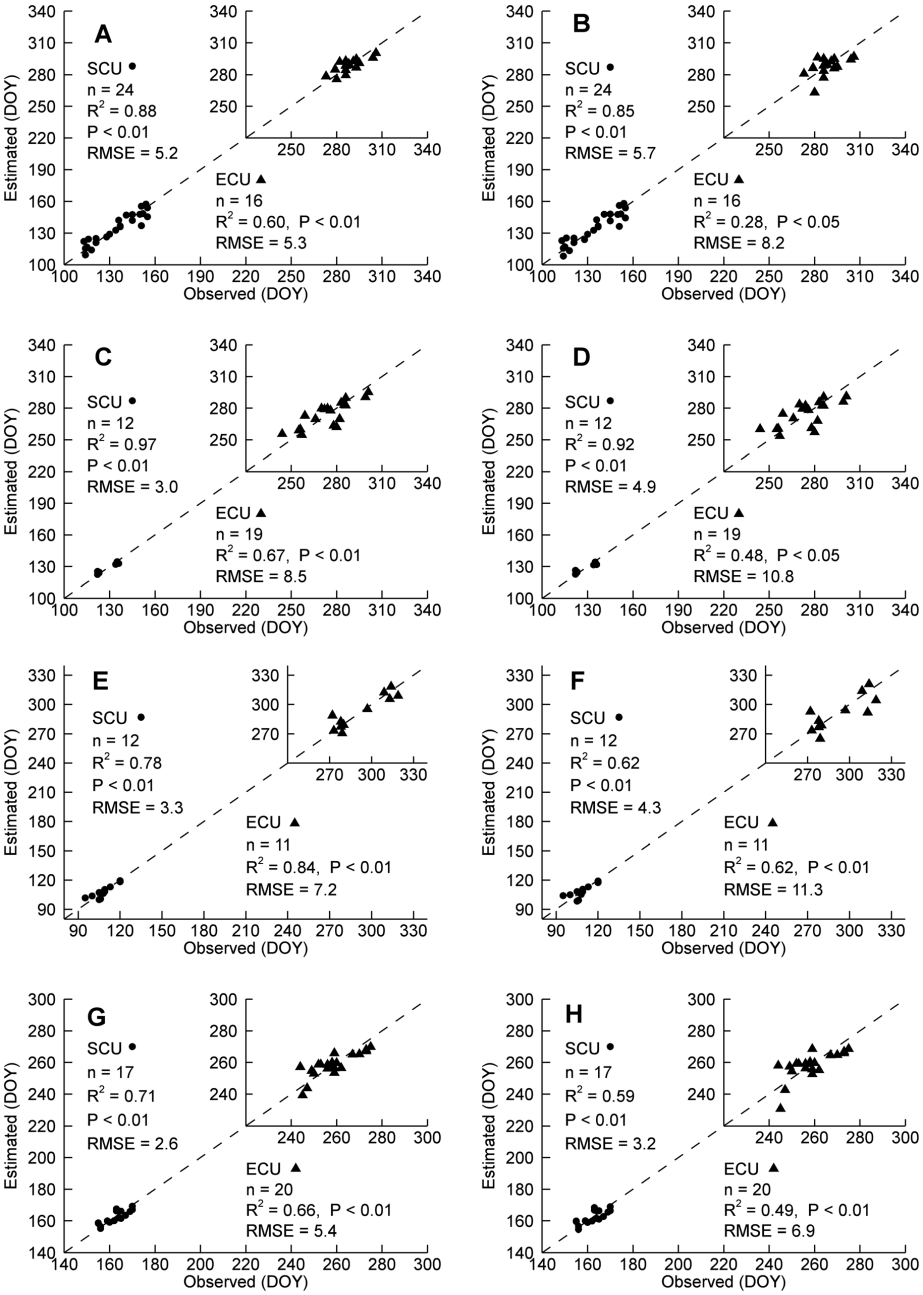
Relationships between observed and estimated carbon flux phenology dates for different biomes. A) and B) Deciduous broadleaf forest, C) and D) Evergreen needleleaf forest, E) and F) Grasslands, and G) and H) Croplands. The left panel (i.e., A, C, E and G) indicates the relationships between observed Start/End of Carbon Uptake (SCU/ECU) in Julian Day of Year (DOY) and estimated with the best performing explanatory variables given in Figure 4, and the right panel (i.e., B, D, F and H) indicates the relationships between observed SCU/ECU and estimated with the best performing explanatory variables based on the leave-one-out cross-validation approach.

Figure 5 showed the relationships between observed and estimated CFP dates based on the linear regression (Figure 5 A, C, E, G) and the leave-one-out cross-validation (Figure 5 B, D, F, H) approaches in terms of the best performing explanatory variables. All of the estimation performances were statistically significant (*P*<0.05). The explained variance for SCU ranged from 71% to 97%, and the RMSE for SCU ranged from 2.6 to 5.2 days (Figure 5 A, C, E, G). Comparing with SCU, the ECU estimation showed a relatively lower performance, with a relatively lower *R* ^2^ (60%–84%) and a higher RMSE (5.3–8.5 days) (Figure 5 A, C, E, G).

The CFP estimation performance varied among different biomes. The estimation RMSE for both SCU and ECU was less than 9 days for deciduous broadleaf forest (Figure 5 A, B). Evergreen needleleaf forest had a lower RMSE for SCU but a higher RMSE for ECU (Figure 5 C, D). Grasslands had the highest *R* ^2^ but also the highest RMSE for ECU (Figure 5 E, F), while croplands had the lowest RMSE for both SCU and ECU (Figure 5 G, H).

## Discussion

### The optimal explanatory variables in estimating CFP dates

The temporal and spatial variation in CFP metrics (i.e., SCU and ECU) is controlled by many factors, including the biome type, canopy structures, species compositions, soil type, forest age and meteorological factors (e.g., temperature, precipitation, etc.) [3], [18], [19], [41]. Wu *et al*. [3] demonstrated that the interannual variation in NEE and phenological indicators at a study site could be mainly resulted from the meteorological factors, while differences of canopy structures and species compositions had no significant impacts. On the contrary, when the spatial variation was considered, the primary controlling factors may be site-specific differences in canopy structures, soil prosperities and biome types. Therefore, they suggested a separated analysis for spatial and temporal variation in the response of annual NEE to CUP and its transitions. In fact, the most challenging aspect in estimating CFP dates is to identify the optimal explanatory variables whether or not the temporal and spatial variation in CFP metrics is separately considered.

The satellite-derived phenological metrics reflect an integrated signal of a group of species (individuals) in a pixel because remote sensing can capture the spectral characteristics of green leaf and vegetation canopy structures at moderate to coarse spatial resolutions [19], [42], [43]. Our results showed high explained SCU/ECU variances with SOS/EOS (Table 1), which have been also found by Gonsamo *et al*. [19]. This suggested that satellite-derived LSP dates can effectively reflect the spatial and temporal variations in CFP dates and should be selected as one of the primary explanatory variables in estimating CFP dates, especially for the large-scale (e.g., regional or continental) studies. However, we should also note that large discrepancies exist in different LSP retrieval methods in terms of the CFP estimating performances (Text S2). Because of the different SOS/EOS definitions (Text S1), the satellite-derived LSP metrics with different methods do not actually measure the same phenological traits [44]. Our results demonstrated that the SCU/ECU is much closer to and more consistent with the time when satellite-derived vegetation index reaches its midpoint in a growing season for the 4 tested biomes.

Meteorological factors are important candidate explanatory variables in estimating CFP dates. Previous results [18], [19] and our results (Table 1, Figure 5) all indicated that combining optimal climate drivers with LSP dates can obviously improve the estimation robustness when the established regression models are applied in a wide spatial and temporal range. Our results also demonstrated the differences in the optimal impact periods of climate drivers on SCU/ECU for different biomes (Figure 4). Therefore, the key question is how to select the optimal explanatory climate drivers for different biomes. Gonsamo *et al*. [19] conducted a sensitivity analysis to search the optimal impact period based on human calendar month (e.g., the mean air temperature in April and May for SCU). Rather than applying the human calendar month, we used the distance (in days) from the LSP dates to identify the optimal impact periods. This is because the LSP dates retrieved with the best performing method are close to the CFP dates and vary simultaneously with CFP dates when across regions and/or biomes, while the unified human calendar month cannot reflect the variations in vegetation phenology across large heterogeneous areas and thus may fail to describe the actual impact period of climate drivers on SCU/ECU.

### The difference in CFP estimation performance among different biomes

Large discrepancies existed in the CFP estimation performance for different biomes. The CFP dates are relatively easy to be estimated for deciduous broadleaf forest because of its distinct seasonal variation in canopy structure and carbon flux which can be effectively captured by remote sensing and eddy covariance system. Our estimated CFP dates based on both LSP dates and climate drivers for deciduous broadleaf forest was comparable with that based on observed carbon flux and meteorology data (5.20 vs. 5.12 for SCU, 5.30 vs. 6.65 for ECU in RMSE) and slightly better than that solely based on satellite data (5.20 vs. 6.98 for SCU, 5.30 vs. 8.88 for ECU in RMSE) from Gonsamo *et al*. [19].

Theoretically, it is difficult to define CFP metrics for conifers because NEE may be negative throughout the year. The transition from net positive to net negative NEE is more flexible in coniferous than in deciduous forest because the seasonality of coniferous forest is not related to changes in canopy structure [45]. However, in high latitude snow-dominated coniferous forests, the annual cycle from near-total snow cover to a mature canopy provides a distinct and detectable VI and NEE cycle [27]. The flux sites for evergreen needleleaf forest in this study are distributed above 45°N and are fully covered by snow in winter. The satellite-derived LSP dates are closely related to the timing of spring snowmelt and winter snow, which substantially reflects the change in air temperature. Therefore, the CFP dates estimation performance with LSP dates and climate drivers for evergreen needleleaf forest in this study was relatively high, especially in estimating SCU. It should be noted that the high estimation performance for evergreen needleleaf forest may be only suitable for snow-dominated ecosystems and cannot be extrapolated to other coniferous forests, such as temperate and subtropical evergreen needleleaf forest.

Out results also demonstrated a high estimation performance for CFP dates of herbaceous biomes (i.e., grassland, crop). The high estimation performance may result from the primary control of satellite-derived LSP dates on estimating CFP dates (Table 1). For example, the phenological development for the 6 selected grassland sites was mainly precipitation-driven (Figure 4 A, B) and their leaf-out and NEE transition was usually occurred in a short period. Moreover, herbaceous biomes do not have understory plants that could confound the spectral signal. Therefore, LSP dates showed a significant variance explanatory rate in estimating CFP dates for herbaceous biomes.

### The difference in estimation performance between SCU and ECU

The SCU estimation performance was generally better than ECU (Figure 5). The explained variance by LSP dates (Table 1) and climate drivers (Figure 4) for ECU was generally lower than that for SCU, implying that the satellite-derived EOS and climate drivers had relatively weak relationships with ECU. During the greenup phase, increasing greenness is closely related to chlorophyll, leaf area and changes in canopy structure, which scale rather well with photosynthesis and respiration [46], [47]. However, during the leaf senescence phase, changes in leaf color, environmental stress (e.g., drought stress), and meteorological conditions (e.g., cooler air temperature) may complicate the relationships between canopy structure-based phenology metrics and carbon fluxes, and in general make the detection of senescence events more difficult [41], [48]. In fact, the factors controlling senescence and dormancy are not well-documented in all biomes [41]. A mechanistic understanding of the drivers controlling senescence and dormancy is urgently needed.

### Potentials of the optimization method in estimating CFP metrics

The optimization method proposed in this study can be used to extrapolate regional CFP metrics through extending the footprints of flux towers. As for a given biome over large heterogeneous areas, a synoptic train of thought is first to classify the biome to smaller ecoregions, or use existing ecoregion maps (e.g., the terrestrial ecoregions compiled by the World Wildlife Fund (WWF) (available at: http://worldwildlife.org/biome-categories/terrestrial-ecoregions)), since vegetation phenology may differ significantly even within the same biome. Then for each ecoregion, time-series satellite data, climate data and NEE data for the involved eddy flux sites can be used to build an optimized empirical model to predict CFP metrics beyond the footprints of flux towers.

## Conclusions

This study provided a methodology to estimate CFP metrics with satellite-derived LSP metrics and climate drivers for different biomes through a synthesis of AmeriFlux observations. LSP metrics combined with optimal climate drivers can explain SCU variability by more than 70% (ranging from 71% to 97% for different biomes) and ECU variability by more than 60% (ranging from 60% to 84% for different biomes). The RMSE of the estimations ranged from 2.6 to 5.2 days for SCU and from 5.3 to 8.5 days for ECU. The results of our study highlighted the relative strengths and weaknesses of LSP metrics derived from different methods and climate drivers in different impact periods in estimating a specific CFP metric for different biomes. The estimation performance for the methodology was primarily dependent on the optimal combination of the LSP retrieval methods, the explanatory climate drivers, the biome types, and the specific CFP metric. Although the proposed methodology showed high performance in estimating CFP metrics for biomes with a distinct and detectable VI and NEE cycle, a better mechanistic understanding of the drivers controlling vegetation phenology is urgently needed in order to improve the estimation performance, especially for senescence and dormancy phenology.

## Supporting Information

Acronym S1 **Acronyms and definitions.**
(DOCX)

Dataset S1 **Eddy flux sites.**
(DOCX)

Text S1 **Supplementary methods.**
(DOCX)

Text S2 **Supplementary results.**
(DOCX)
