# Supplementary material for: Estimating Carbon Flux Phenology with Satellite-Derived Land Surface Phenology and Climate Drivers for Different Biomes: A Synthesis of AmeriFlux Observations
Source: PLoS One. 2013 Dec 27;8(12):e84990. doi: 10.1371/journal.pone.0084990 (PMC3873994; doi:10.1371/journal.pone.0084990)
Supplement: Acronym S1 — Acronyms and definitions. (DOCX) [file pone.0084990.s001.docx]

Acronym S1 Acronyms and definitions

| Acronym | Definition |
| --- | --- |
| CFP | Carbon Flux Phenology |
| CUP | Carbon Uptake Period |
| DOY | Julian Day of Year |
| ECU | End of Carbon Uptake |
| EOS | End of Season |
| EVI | Enhanced Vegetation Index |
| GSL | Growing Season Length |
| IGBP | International Geosphere-Biosphere Program |
| LSP | Land Surface Phenology |
| MODIS | Moderate Resolution Imaging Spectroradiometer |
| NDVI | Normalized Difference Vegetation Index |
| NEE | Net Ecosystem Exchange of carbon |
| RMSE | Root Mean Square Error |
| SCU | Start of Carbon Uptake |
| SOS | Start of Season |
| VI | Vegetation Index |
