# Supplementary material for: Estimating Carbon Flux Phenology with Satellite-Derived Land Surface Phenology and Climate Drivers for Different Biomes: A Synthesis of AmeriFlux Observations
Source: PLoS One. 2013 Dec 27;8(12):e84990. doi: 10.1371/journal.pone.0084990 (PMC3873994; doi:10.1371/journal.pone.0084990)
Supplement: Dataset S1 — Eddy flux sites. (DOCX) [file pone.0084990.s002.docx]

Dataset S2 AmeriFlux eddy flux sites

| Site full name | Site ID | Latitude | Longitude | IGBP Classification |
| --- | --- | --- | --- | --- |
| ARM SGP Burn | US-ArB | 35.549740 | -98.040230 | Grasslands |
| ARM SGP Control | US-ArC | 35.546490 | -98.040060 | Grasslands |
| Bartlett Experimental Forest | US-Bar | 44.064640 | -71.288077 | Deciduous Broadleaf Forest |
| Bondville | US-Bo1 | 40.006100 | -88.291867 | Croplands |
| Brookings | US-Bkg | 44.345290 | -96.836170 | Grasslands |
| Fort Peck | US-FPe | 48.307700 | -105.101900 | Grasslands |
| Goodwin Creek | US-Goo | 34.250000 | -89.970569 | Grasslands |
| Harvard Forest | US-Ha1 | 42.537756 | -72.171478 | Deciduous Broadleaf Forest |
| Howland Forest Main | US-Ho1 | 45.204100 | -68.740200 | Evergreen Needleleaf Forest |
| Howland Forest West Tower | US-Ho2 | 45.209120 | -68.747000 | Evergreen Needleleaf Forest |
| Mead Irrigated | US-Ne1 | 41.165056 | -96.476638 | Croplands |
| Mead Irrigated Rotation | US-Ne2 | 41.164871 | -96.470100 | Croplands |
| Mead Rainfed | US-Ne3 | 41.179667 | -96.439646 | Croplands |
| Missouri Ozark | US-MOz | 38.744110 | -92.200009 | Deciduous Broadleaf Forest |
| Morgan Monroe State Forest | US-MMS | 39.323150 | -86.413139 | Deciduous Broadleaf Forest |
| Ohio Oak Openings | US-Oho | 41.554540 | -83.843760 | Deciduous Broadleaf Forest |
| UCI 1850 | CA-NS1 | 55.879167 | -98.483889 | Evergreen Needleleaf Forest |
| UCI 1930 | CA-NS2 | 55.905833 | -98.524722 | Evergreen Needleleaf Forest |
| UCI 1964 | CA-NS3 | 55.911667 | -98.382222 | Evergreen Needleleaf Forest |
| UCI 1964wet | CA-NS4 | 55.911667 | -98.382222 | Evergreen Needleleaf Forest |
| UCI 1981 | CA-NS5 | 55.863056 | -98.485000 | Evergreen Needleleaf Forest |
| UCI 1989 | CA-NS6 | 55.916667 | -98.964444 | Evergreen Needleleaf Forest |
| UCI 1998 | CA-NS7 | 56.635833 | -99.948333 | Evergreen Needleleaf Forest |
| UMBS | US-UMB | 45.559840 | -84.713820 | Deciduous Broadleaf Forest |
| Walnut River | US-Wlr | 37.520833 | -96.855000 | Grasslands |
| Willow Creek | US-WCr | 45.805927 | -90.079859 | Deciduous Broadleaf Forest |
| Wisconsin Clearcut Young Hardwood | US-Wi8 | 46.722333 | -91.252417 | Deciduous Broadleaf Forest |
| Wisconsin Intermediate Hardwoods | US-Wi1 | 46.730472 | -91.232944 | Deciduous Broadleaf Forest |
| Wisconsin Intermediate Red Pine | US-Wi2 | 46.686889 | -91.152833 | Evergreen Needleleaf Forest |
| Wisconsin Mixed Young Jack Pine | US-Wi5 | 46.653083 | -91.085806 | Evergreen Needleleaf Forest |
| Wisconsin Young Jack Pine | US-Wi9 | 46.618778 | -91.081444 | Evergreen Needleleaf Forest |
| Wisconsin Young Red Pine | US-Wi0 | 46.618778 | -91.081444 | Evergreen Needleleaf Forest |
