# Supplementary material for: Estimating Carbon Flux Phenology with Satellite-Derived Land Surface Phenology and Climate Drivers for Different Biomes: A Synthesis of AmeriFlux Observations
Source: PLoS One. 2013 Dec 27;8(12):e84990. doi: 10.1371/journal.pone.0084990 (PMC3873994; doi:10.1371/journal.pone.0084990)
Supplement: Text S1 — Supplementary methods. (DOCX) [file pone.0084990.s003.docx]

Text S3 Supplementary methods

Satellite-based methods for retrieving Land Surface Phenology (LSP) dates. There are 3 types of satellite-based LSP retrieval methods, including the threshold method (i.e., a global absolute threshold value or a local relative threshold value defined as a fraction of the annual amplitude) [1-4], the autoregressive moving average method [5,6] and the function fitting method [7-12]. The global threshold method ignores the differences in the NDVI values in different regions or different biomes. Consequently, White *et al*. [4] proposed a local midpoint threshold method to identify the onset date of the vegetation growth season in the United States of America during 1990-1992. The autoregressive moving average method was first proposed by Reed *et al*. [6] to measure phenological variability from satellite imagery, and the derived phenological metrics with this method have been proven to be consistent with the ground observed phenological events [5,6]. For the function fitting method, many mathematical functions, such as the asymmetric Gaussian function [8], the polynomial function [9], the piecewise logistic function [10-12] and the double logistic function [7], are used to fit the VI time series. Almost all the methods mentioned above have been proven to be consistent with their given references (e.g., ground observed phenology events, model simulated vegetation phenology or eddy covariance flux tower-derived phenological metrics), but it was very difficult to give the ordinal rank of Start of Season (SOS) methods because they varied geographically [5]. Therefore, this paper first investigated these 3 types of satellite methods (including 6 specific retrieval methods) for retrieving LSP dates, and then selected the best one to retrieve LSP dates as explanatory variables in estimating Carbon Flux Phenology (CFP) dates. The detailed descriptions of these 6 specific retrieval methods were shown below.

For the threshold method, SOS (End of Season, EOS) is defined as the Julian Day of Year (DOY) when the Vegetation Index (VI) curve crosses the threshold in an upward (downward) phase. According to previous studies [1-3,5], the global threshold method is known to be not appropriate for large scale mapping of SOS and EOS [4]. Therefore, we only applied two local threshold methods. For the first local threshold method, the local threshold was set as the midpoint between the minimum and the maximum values of the VI time series in a growth cycle for each year (We call it the local midpoint threshold method hereafter). The other method is similar to the first local threshold method, except that the local midpoint threshold is derived from a multi-year average VI time series (We call it the local mean midpoint threshold method hereafter) [4].

For the autoregressive moving average method, SOS (EOS) is defined as the DOY when the VI curve crosses the moving average time series in an upward (downward) phase [6]. The time interval is a critical parameter for this method. Reed *et al*. [6] set the time interval as 9 for a 14-day composite Normalized Difference Vegetation Index (NDVI) time series after testing several time intervals, varying from 3 to 15. White *et al*. [5] set the time interval as 15 for a 15-day composite NDVI time series. The NDVI data involved in this study was a 16-day composite product, so we set the time interval as 15 according to White *et al*. [5].

For the function fitting method, SOS and EOS is extracted based on the characteristics of the mathematical function that is used to fit the VI time series. In this study, we selected the Logistic function and the polynomial function as the mathematical fitting functions. For the Logistic function method, there are two algorithms to retrieve SOS/EOS: (1) in the first derivative algorithm, SOS is defined as the maximum of the first derivative of the fitted Logistic function (indicating a maximum VI increase) and EOS is defined as the minimum of the first derivative (indicating a maximum VI decrease) [5]; (2) in the change rate of the curvature algorithm, vegetation transition dates (e.g., greenup, maturity, senescence and dormancy) are determined by the maximum or minimum of the change rate of curvature, which is derived from the fitted Logistic function [10]. For the polynomial function fitting method, we first determined the thresholds for SOS and EOS based on the mean VI curve, which is obtained by averaging several growing seasons for each pixel. Then, the VI time series are fitted using a 6-degree polynomial function, and SOS (EOS) is defined as the DOY when the fitted curve crosses the determined threshold [9].

Evaluation of satellite-based LSP retrieval methods. The coefficient of determination (*R*^2^), Root Mean Square Error (RMSE) and Bias were used to evaluate the performances of the satellite-based LSP retrieval methods, based on the observed Carbon Flux Phenology (CFP) dates for different biomes. The coefficient of determination can be used to explain the CFP variance. A higher *R*^2^ means a higher explained variance. The RMSE is a combined measure of the bias and variance that is associated with an estimator [13]. A lower RMSE indicates a smaller discrepancy between the LSP and CFP dates. The bias shows the number of days that the satellite-retrieved LSP dates deviates from the carbon flux-derived CFP dates. A positive/negative bias indicates a late/early LSP dates relative to the CFP dates. The closer the bias to 0, the smaller is the difference between the LSP and CFP dates. Significance test for the coefficient of determination was conducted by *F*-test with the standard 0.05 cutoff indicating statistical significance (i.e., *P* < 0.05).

References

1. Lloyd D (1990) A phenological classification of terrestrial vegetation cover using shortwave vegetation index imagery. International Journal of Remote Sensing 11: 2269-2279.

2. Fischer A (1994) A model for the seasonal variations of vegetation indices in coarse resolution data and its inversion to extract crop parameters. Remote Sensing of Environment 48: 220-230.

3. Markon CJ, Fleming MD, Binnian EF (1995) Characteristics of vegetation phenology over the Alaskan landscape using AVHRR time-series data. Polar Record 31: 179-190.

4. White MA, Thornton PE, Running SW (1997) A continental phenology model for monitoring vegetation responses to interannual climatic variability. Global Biogeochemical Cycles 11: 217-234.

5. White MA, Beurs KM, Didan K, Inouye DW, Richardson AD, et al. (2009) Intercomparison, interpretation, and assessment of spring phenology in North America estimated from remote sensing for 1982-2006. Global Change Biology 15: 2335-2359.

6. Reed BC, Brown JF, VanderZee D, Loveland TR, Merchant JW, et al. (1994) Measuring phenological variability from satellite imagery. Journal of Vegetation Science 5: 703-714.

7. Beck PSA, Atzberger C, Hogda KA, Johansen B, Skidmore AK (2006) Improved monitoring of vegetation dynamics at very high latitudes: A new method using MODIS NDVI. Remote Sensing of Environment 100: 321-334.

8. Jönsson P, Eklundh L (2002) Seasonality extraction by function fitting to time-series of satellite sensor data. IEEE Transactions on Geoscience and Remote Sensing 40: 1824-1832.

9. Piao SL, Fang JY, Zhou LM, Ciais P, Zhu B (2006) Variations in satellite-derived phenology in China's temperate vegetation. Global Change Biology 12: 672-685.

10. Zhang XY, Friedl MA, Schaaf CB, Strahler AH, Hodges J, et al. (2003) Monitoring vegetation phenology using MODIS. Remote Sensing of Environment 84: 471-475.

11. Zhang XY, Friedl MA, Schaaf CB, Strahler AH (2004) Climate controls on vegetation phenological patterns in northern mid- and high latitudes inferred from MODIS data. Global Change Biology 10: 1133-1145.

12. Zhu W, Tian H, Xu X, Pan Y, Chen G, et al. (2012) Extension of the growing season due to delayed autumn over mid and high latitudes in North America during 1982–2006. Global Ecology and Biogeography 21: 260-271.

13. Lobell DB, Asner GP (2004) Cropland distributions from temporal unmixing of MODIS data. Remote Sensing of Environment 93: 412-422.
