# Supplementary material for: Estimating Carbon Flux Phenology with Satellite-Derived Land Surface Phenology and Climate Drivers for Different Biomes: A Synthesis of AmeriFlux Observations
Source: PLoS One. 2013 Dec 27;8(12):e84990. doi: 10.1371/journal.pone.0084990 (PMC3873994; doi:10.1371/journal.pone.0084990)
Supplement: Text S2 — Supplementary results. (DOCX) [file pone.0084990.s004.docx]

Text S4 Supplementary results

Performance of the satellite-based Land Surface Phenology (LSP) retrieval methods

There were 6 satellite-based LSP retrieval methods been used to identify the best-performing method, including local midpoint threshold, local mean midpoint threshold, autoregressive moving average, change rate of curvature, first derivative and polynomial function fitting. Table S1-6 listed the performance for each method based on the Normalized Difference Vegetation Index (NDVI) time series. There were large discrepancies among different methods regarding the Carbon Flux Phenology (CFP) estimating performance. For example, the coefficient of determination (*R*^2^) between carbon flux-derived Start of Carbon Uptake (SCU) and satellite-derived Start of Season (SOS) ranged from 1.2% to 75.8% for deciduous broadleaf forest, from 0.5% to 78.4% for evergreen needleleaf forest, from 3.3% to 43.1% for grasslands and from 1.8% to 68.8% for croplands. Among these 6 methods, the local mean midpoint threshold method showed the best performances with a higher *R*^2^, lower RMSE and bias for both a single biome and all biomes (Table S2). Therefore, the LSP dates retrieved with the local mean midpoint threshold method were used to estimate CFP dates.

EVI vs. NDVI

Figure S1 presented the comparison between Normalized Difference Vegetation Index (NDVI) and Enhance Vegetation Index (EVI) for the estimation of Start/End of Season (SOS/EOS) based on the local mean midpoint threshold method. In fact, the timing of SOS (EOS) was located at the middle of the linearly increasing (decreasing) part of both EVI and NDVI time-series curves. This meant that the extremely low or high values in the NDVI time series were not involved in the SOS/EOS extraction, which avoided the issues arising from the soil background effects for low NDVI values and the saturation effects for high NDVI values. Moreover, NDVI values close to the SOS/EOS were more sensitive to vegetation growth than EVI (Table S7), which implied that NDVI may be more suitable for SOS/EOS estimation than EVI. In fact, the SOS/EOS estimation accuracy showed that NDVI was distinctly superior to EVI in extracting SOS/EOS when referring to the same Net Ecosystem Exchange (NEE)-derived carbon flux phenology dates (Table S2 vs. Table S8). For example, EVI can get a higher Coefficient of determination (*R*^2^) between SOS and the Start of Carbon Uptake (SCU) than NDVI for deciduous broadleaf forest, while NDVI was superior to EVI for evergreen needleleaf forest and cropland. As for EOS, NDVI could obtain a far higher *R*^2^ than EVI for all biome types. Therefore, we selected NDVI but not EVI to extract SOS/EOS.

Table S1. The performance for the local midpoint threshold method based on the Normalized Difference Vegetation Index (NDVI) time-series data

| Biome type | SCU vs. SOS^†^ | | | | ECU vs. EOS^†^ | | | |
| --- | --- | --- | --- | --- | --- | --- | --- | --- |
|  | Samples | *R*^2^ (%) | RMSE | Bias | Samples | *R*^2^ (%) | RMSE | Bias |
| Deciduous broadleaf forest | 24 | 75.8^*^ | 7.3 | -11.5 | 20 | 58.0^*^ | 5.9 | 8.0 |
| Evergreen needleleaf forest | 16 | 16.3 | 15.1 | -0.1 | 30 | 29.7^*^ | 14.6 | 38.9 |
| Grasslands | 16 | 17.5 | 7.9 | 2.8 | 14 | 76.6^*^ | 8.7 | 8.6 |
| Croplands | 17 | 27.9^*^ | 4.2 | -2.3 | 22 | 45.2^*^ | 7.0 | 15.9 |
| All biomes | 73 | 58.4^*^ | 15.5 | -3.8 | 86 | 38.2^*^ | 15.3 | 20.9 |

^†^ *R*^2^=Coefficient of determination, RMSE=Root Mean Square Error, SCU=Start of Carbon Uptake derived from carbon flux data, SOS=Start of Season derived from satellite data, ECU=End of Carbon Uptake derived from carbon flux data, EOS=End of Season derived from satellite data.

^*^ Statistically significant at the 0.05 level.

Table S2. The performance for the local mean midpoint threshold method based on the Normalized Difference Vegetation Index (NDVI) time-series data

| Biome type | SCU vs. SOS^†^ | | | | ECU vs. EOS^†^ | | | |
| --- | --- | --- | --- | --- | --- | --- | --- | --- |
|  | Samples | *R*^2^ (%) | RMSE | Bias | Samples | *R*^2^ (%) | RMSE | Bias |
| Deciduous broadleaf forest | 24 | 74.3^*^ | 7.5 | -10.5 | 20 | 51.4^*^ | 6.3 | 4.6 |
| Evergreen needleleaf forest | 16 | 78.4^*^ | 7.6 | 15.8 | 30 | 43.5^*^ | 13.1 | 35.6 |
| Grasslands | 16 | 43.1^*^ | 6.5 | 3.3 | 14 | 67.1^*^ | 10.3 | 9.4 |
| Croplands | 17 | 68.8^*^ | 2.7 | -0.2 | 22 | 65.0^*^ | 5.6 | 14.7 |
| All biomes | 73 | 49.6^*^ | 17.1 | 0.7 | 86 | 43.5^*^ | 14.6 | 18.8 |

^†^ *R*^2^=Coefficient of determination, RMSE=Root Mean Square Error, SCU=Start of Carbon Uptake derived from carbon flux data, SOS=Start of Season derived from satellite data, ECU=End of Carbon Uptake derived from carbon flux data, EOS=End of Season derived from satellite data.

^*^ Statistically significant at the 0.05 level.

Table S3. The performance for the autoregressive moving average method based on the Normalized Difference Vegetation Index (NDVI) time-series data

| Biome type | SCU vs. SOS^†^ | | | | ECU vs. EOS^†^ | | | |
| --- | --- | --- | --- | --- | --- | --- | --- | --- |
|  | Samples | *R*^2^ (%) | RMSE | Bias | Samples | *R*^2^ (%) | RMSE | Bias |
| Deciduous broadleaf forest | 24 | 74.6^*^ | 7.5 | -17.9 | 20 | 4.0 | 8.9 | 37.8 |
| Evergreen needleleaf forest | 16 | 64.8^*^ | 9.8 | 10.9 | 30 | 37.0^*^ | 13.9 | 62.6 |
| Grasslands | 16 | 29.6^*^ | 7.3 | -2.9 | 14 | 55.1^*^ | 12.0 | 33.3 |
| Croplands | 17 | 63.2^*^ | 3.0 | -35.9 | 22 | 48.0^*^ | 6.9 | 67.5 |
| All biomes | 73 | 17.2^*^ | 21.9 | -12.5 | 86 | 15.4^*^ | 17.8 | 53.3 |

^†^ *R*^2^=Coefficient of determination, RMSE=Root Mean Square Error, SCU=Start of Carbon Uptake derived from carbon flux data, SOS=Start of Season derived from satellite data, ECU=End of Carbon Uptake derived from carbon flux data, EOS=End of Season derived from satellite data.

^*^ Statistically significant at the 0.05 level.

Table S4. The performance for the change rate of curvature method based on the Normalized Difference Vegetation Index (NDVI) time-series data

| Biome type | SCU vs. SOS^†^ | | | | ECU vs. EOS^†^ | | | |
| --- | --- | --- | --- | --- | --- | --- | --- | --- |
|  | Samples | *R*^2^ (%) | RMSE | Bias | Samples | *R*^2^ (%) | RMSE | Bias |
| Deciduous broadleaf forest | 24 | 1.2 | 14.8 | -48.8 | 20 | 30.2^*^ | 7.6 | 63.0 |
| Ever green needleleaf forest | 16 | 0.5 | 16.4 | -38.1 | 30 | 19.9^*^ | 15.6 | 84.3 |
| Grasslands | 16 | 9.5 | 8.2 | -40.2 | 14 | 33.5^*^ | 14.6 | 69.6 |
| Croplands | 17 | 1.8 | 4.9 | -38.6 | 22 | 5.5 | 9.2 | 69.7 |
| All biomes | 73 | 27.8^*^ | 20.5 | -42.2 | 86 | 37.0^*^ | 15.4 | 73.2 |

^†^ *R*^2^=Coefficient of determination, RMSE=Root Mean Square Error, SCU=Start of Carbon Uptake derived from carbon flux data, SOS=Start of Season derived from satellite data, ECU=End of Carbon Uptake derived from carbon flux data, EOS=End of Season derived from satellite data.

^*^ Statistically significant at the 0.05 level.

Table S5. The performance for the first derivative method based on the Normalized Difference Vegetation Index (NDVI) time-series data

| Biome type | SCU vs. SOS^†^ | | | | ECU vs. EOS^†^ | | | |
| --- | --- | --- | --- | --- | --- | --- | --- | --- |
|  | Samples | *R*^2^ (%) | RMSE | Bias | Samples | *R*^2^ (%) | RMSE | Bias |
| Deciduous broadleaf forest | 24 | 68.2^*^ | 8.4 | -4.7 | 20 | 46.0^*^ | 6.6 | 15.5 |
| Evergreen needleleaf forest | 16 | 2.7 | 16.2 | 20.7 | 30 | 40.3^*^ | 13.5 | 41.7 |
| Grasslands | 16 | 16.1 | 7.9 | 5.2 | 14 | 73.9^*^ | 9.2 | 22.4 |
| Croplands | 17 | 41.2^*^ | 3.8 | 10.1 | 22 | 60.9^*^ | 5.9 | 22.7 |
| All biomes | 73 | 50.1^*^ | 17.0 | 6.5 | 86 | 51.6^*^ | 13.5 | 27.6 |

^†^ *R*^2^=Coefficient of determination, RMSE=Root Mean Square Error, SCU=Start of Carbon Uptake derived from carbon flux data, SOS=Start of Season derived from satellite data, ECU=End of Carbon Uptake derived from carbon flux data, EOS=End of Season derived from satellite data.

^*^ Statistically significant at the 0.05 level.

Table S6. The performance for the polynomial function fitting method based on the Normalized Difference Vegetation Index (NDVI) time-series data

| Biome type | SCU vs. SOS^†^ | | | | ECU vs. EOS^†^ | | | |
| --- | --- | --- | --- | --- | --- | --- | --- | --- |
|  | Samples | *R*^2^ (%) | RMSE | Bias | Samples | *R*^2^ (%) | RMSE | Bias |
| Deciduous broadleaf forest | 24 | 12.7 | 13.9 | -33.4 | 20 | 46.0^*^ | 6.6 | 19.3 |
| Evergreen needleleaf forest | 16 | 63.6^*^ | 9.9 | -0.3 | 30 | 2.4 | 17.2 | 76.5 |
| Grasslands | 16 | 3.3 | 8.5 | -21.4 | 14 | 75.8^*^ | 8.8 | 41.9 |
| Croplands | 17 | 27.5^*^ | 4.2 | 1.3 | 22 | 39.3^*^ | 7.4 | 40.7 |
| All biomes | 73 | 17.3^*^ | 21.9 | -15.5 | 86 | 15.1^*^ | 17.9 | 48.4 |

^†^ *R*^2^=Coefficient of determination, RMSE=Root Mean Square Error, SCU=Start of Carbon Uptake derived from carbon flux data, SOS=Start of Season derived from satellite data, ECU=End of Carbon Uptake derived from carbon flux data, EOS=End of Season derived from satellite data.

^*^ Statistically significant at the 0.05 level.


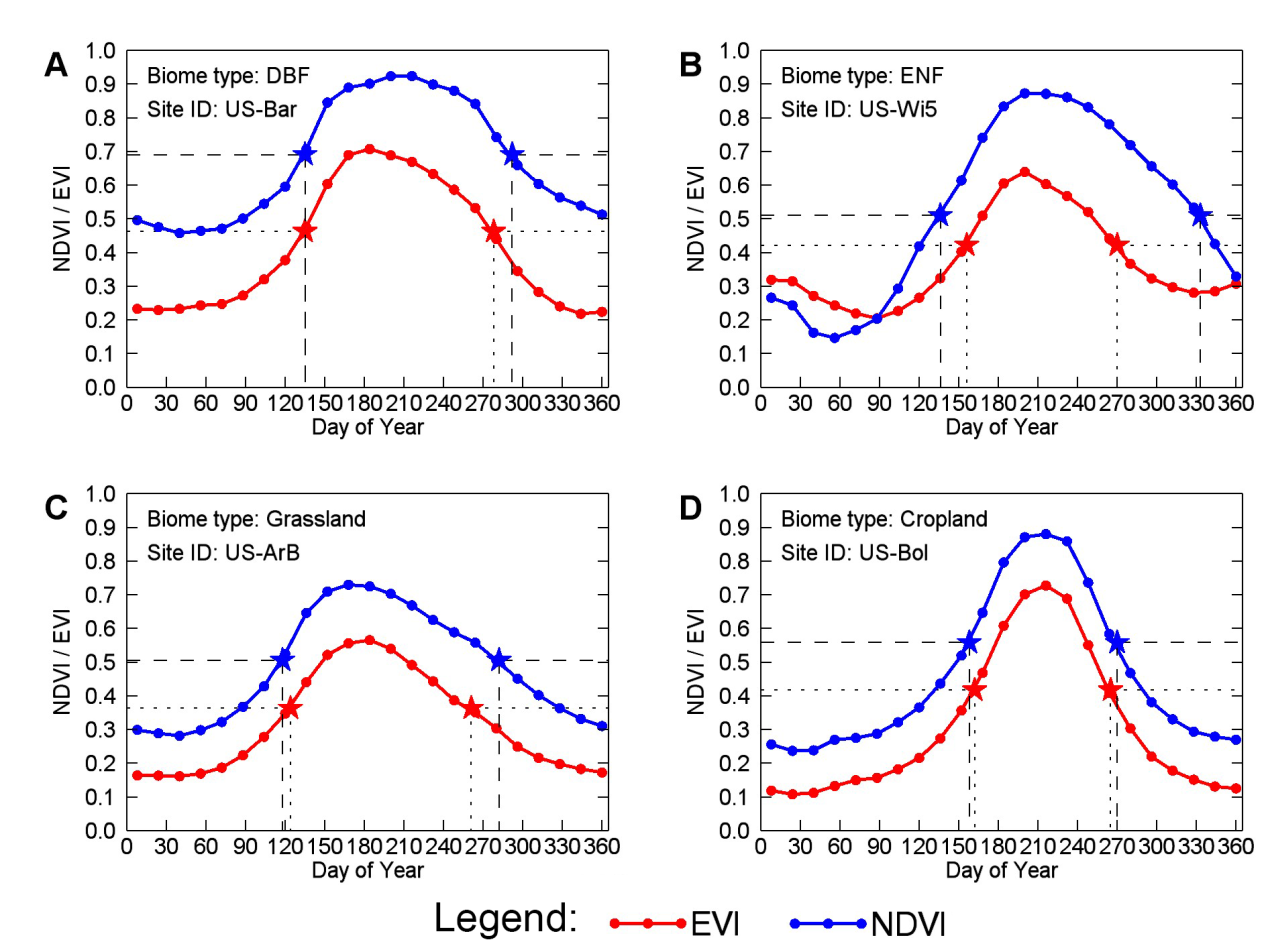


Figure S1. Comparison between Normalized Difference Vegetation Index (NDVI) and Enhanced Vegetation Index (EVI) for the estimation of Start/End of Season (SOS/EOS) based on the local mean midpoint threshold method. EVI/NDVI time series were temporally averaged during 2000-2008 for each site. For each EVI/NDVI time series, the star symbol in spring indicates the timing of SOS, and the star in autumn indicates the timing of EOS. Note that the timing of SOS (EOS) is located at the middle of the linearly increasing (decreasing) part of the EVI/NDVI time-series curve.

Table S7. The sensitivity (1/day) of Normalized Difference Vegetation Index (NDVI) and Enhanced Vegetation Index (EVI) to vegetation growth in the period between 20-day before and 20-day after SOS/EOS^§^.

| Biome type | DBF^†^ | | ENF^†^ | | Grassland | | Cropland | |
| --- | --- | --- | --- | --- | --- | --- | --- | --- |
| Site ID | US-Bar | | US-Wi5 | | US-ArB | | US-Bo1 | |
| VI^†^ | EVI | NDVI | EVI | NDVI | EVI | NDVI | EVI | NDVI |
| SOS^†^ | 0.0068 | 0.0074 | 0.0060 | 0.0062 | 0.0052 | 0.0065 | 0.0072 | 0.0074 |
| EOS^†^ | -0.0057 | -0.0047 | -0.0044 | -0.0059 | -0.0026 | -0.0033 | -0.0076 | -0.0076 |

^§^ The sensitivity of EVI/NDVI to vegetation growth was expressed as the linearly regressed slope of EVI/NDVI against the Julian day of year.

^†^ DBF=Deciduous Broadleaf Forest, ENF=Evergreen Needleleaf Forest, VI=Vegetation Index, SOS=Start of Season derived from satellite data, EOS=End of Season derived from satellite data.

Table S8. The performances for the local mean midpoint threshold method based on the Enhanced Vegetation Index (EVI) data.

| Biome type | SCU vs. SOS^†^ | | | | ECU vs. EOS^†^ | | | |
| --- | --- | --- | --- | --- | --- | --- | --- | --- |
|  | Samples | *R*^2^ (%) | RMSE | Bias | Samples | *R*^2^ (%) | RMSE | Bias |
| Deciduous broadleaf forest | 24 | 91.3^*^ | 4.4 | -4.4 | 20 | 25.0^*^ | 7.8 | -9.0 |
| Evergreen needleleaf forest | 16 | 51.3^*^ | 11.5 | -35.7 | 30 | 2.0 | 17.3 | -12.4 |
| Grasslands | 16 | 65.7^*^ | 5.1 | 7.0 | 14 | 34.9^*^ | 14.5 | -9.8 |
| Croplands | 17 | 65.2^*^ | 2.9 | 1.5 | 22 | 21.6^*^ | 8.4 | 9.8 |
| All biomes | 73 | 13.1^*^ | 22.4 | -7.4 | 86 | 5.6^*^ | 18.8 | -5.5 |

^†^ *R*^2^=Coefficient of determination, RMSE=Root Mean Square Error, SCU=Start of Carbon Uptake derived from carbon flux data, SOS=Start of Season derived from satellite data, ECU=End of Carbon Uptake derived from carbon flux data, EOS=End of Season derived from satellite data.

^*^ Statistically significant at the 0.05 level.
